# The Lessons of the Measles Epidemic

**Published:** 1912-08-24

**Authors:** 


### THE LESSONS OF THE MEASLES EPIDEMIC.

Additional interest is this year to be found in the recently issued fourteenth Annual Report of the Metropolitan Asylums Board, inasmuch as this is the Report containing the statistics relating to the epidemic of measles which visited the Metropolis in the winter and spring of last year. By an order dated February 18, 1911, the Local Government Board sanctioned the admission to any of the Metropolitan Asylums Board hospitals of poor children suffering from measles or whooping-cough received through the Poor-Law authorities. At the end of May this order was extended to include, subject to certain restrictions, the admission of non-pauper cases of measles. As a result there were admitted into the Board hospitals 3,144 cases of measles during 1911. Seeing that these cases represent an enormous majority of young children and practically a selection of the worst cases of this illness taken from the poorest and most unsanitary houses in London, it must be admitted that a death-rate of 13.89 per cent. is by no means unsatisfactory. As might be expected, the death-rate was highest among the cases received into the Eastern Hospital—namely, 20.47 per cent. of 743 cases—and lowest at the Western Hospital, 10 per cent. of 400 cases. These figures are very significant, and should give an impressive lesson to those persons who habitually regard measles as a trivial illness. It is well to contrast this with the present death-rate of scarlet-fever cases, which is just under 2 per cent. for the 8,818 cases admitted last year. As a further example of the effect of environment, social status, and age on the mortality rate of measles, we may compare the figures of the London Fever Hospital with those of the Eastern and Western Hospitals referred to above. Of some 114 cases admitted to the London Fever Hospital last year only two died, but then the ages of 84 of these were between 15 and 29 years, and it must be remembered that all the patients of this hospital pay or are paid for. It is noteworthy that of the 3,000 cases admitted into the Board hospitals only 25 were between the above ages, none of whom died.

From the returns of the Registrar-General it appears that in London for the week ending March 18 the number of deaths was the highest recorded since the establishment of civil registration in 1838, and that the epidemic wave began to rise as far back as July 1910, though not until October was there any very noticeable increase in the average number of deaths. The wave fell very much more abruptly than it rose, and from May onwards the number of deaths did not exceed the average, while since November they have been considerably below. During the whole of the year there were recorded 2,570 deaths. It is very difficult to estimate how many cases these deaths represent, as except in one borough measles was not notifiable, and naturally the fatality of this disease for the whole of London would be far below that of the class of case admitted to hospitals. Roughly speaking, the total number of deaths from measles might represent from forty to fifty thousand cases. As usual, the vast majority of the deaths were due to bronchitis or broncho-pneumonia. As regards geographical distribution, the largest number of admissions came from Wandsworth, followed at some distance by those from Poplar and Hackney; curiously enough there was but a small number admitted from Bermondsey. During the early part of last year the disease known as rubella, or German measles, was also very prevalent, and it is interesting to note that of 417 cases gaining admission to the Board hospitals nearly half were certified as scarlet-fever cases and over half as measles.

